# Reliability and Validity of a Survey of Cat Caregivers on Their Cats’ Socialization Level in the Cat’s Normal Environment

**DOI:** 10.3390/ani3041194

**Published:** 2013-12-18

**Authors:** Margaret Slater, Laurie Garrison, Katherine Miller, Emily Weiss, Kathleen Makolinski, Natasha Drain

**Affiliations:** 1 Shelter Research and Development, Community Outreach, American Society for the Prevention of Cruelty to Animals (ASPCA^®^), 50 Stone Ridge Drive, Florence, MA 01062, USA; 2 Shelter Research and Development, Community Outreach, American Society for the Prevention of Cruelty to Animals (ASPCA^®^), P.O. Box 408, Little Silver, NJ 07739, USA; E-Mail: laurie.garrison@aspca.org; 3 Shelter Research and Development, Community Outreach, American Society for the Prevention of Cruelty to Animals (ASPCA^®^), 520 Eighth Avenue, 7th Floor, New York, NY 10018, USA; E-Mail: katherine.miller@aspca.org; 4 Shelter Research and Development, Community Outreach, American Society for the Prevention of Cruelty to Animals (ASPCA^®^), 3201 SW Winding Way, Palm City, FL 34990, USA; E-Mail: emily.weiss@aspca.org; 5 Veterinary Outreach, Community Outreach, American Society for the Prevention of Cruelty to Animals (ASPCA^®^), P.O. Box 1144, Orchard Park, NY 14127, USA; E-Mail: kathleen.makolinski@aspca.org; 6 Shelter Research and Development, Community Outreach, American Society for the Prevention of Cruelty to Animals (ASPCA^®^), P.O. Box 4323, Arlington, VA 22204, USA; E-Mail: natasha.drain@aspca.org

**Keywords:** feral cat, behavior evaluation, stray cat, socialization, survey, reliability, validity

## Abstract

**Simple Summary:**

Many animal welfare organizations accept cats with no known behavioral history. It can be difficult to differentiate between a frightened but well-socialized cat and an unsocialized cat in an animal shelter environment. Making this distinction can save lives, yet currently there is no valid tool. Here we measured the quality of a survey designed to determine socialization level using information from the cat’s caregiver about a cat’s usual behavior around people in the cat’s normal environment. This survey will be used to help develop an effective process that accurately differentiates cats by their socialization levels in animal shelters.

**Abstract:**

Stray cats routinely enter animal welfare organizations each year and shelters are challenged with determining the level of human socialization these cats may possess as quickly as possible. However, there is currently no standard process to guide this determination. This study describes the development and validation of a caregiver survey designed to be filled out by a cat’s caregiver so it accurately describes a cat’s personality, background, and full range of behavior with people when in its normal environment. The results from this survey provided the basis for a socialization score that ranged from unsocialized to well socialized with people. The quality of the survey was evaluated based on inter-rater and test-retest reliability and internal consistency and estimates of construct and criterion validity. In general, our results showed moderate to high levels of inter-rater (median of 0.803, range 0.211–0.957) and test-retest agreement (median 0.92, range 0.211–0.999). Cronbach’s alpha showed high internal consistency (0.962). Estimates of validity did not highlight any major shortcomings. This survey will be used to develop and validate an effective assessment process that accurately differentiates cats by their socialization levels towards humans based on direct observation of cats’ behavior in an animal shelter.

## 1. Introduction

Millions of cats enter animal shelters each year in the United States [1,2]. Cats are among the species most at risk for euthanasia in shelters, with nationwide euthanasia rates of approximately 70% [3]. While many of these cats are relinquished by their owners, at least half enter the shelter as strays with no known history [4,5,6]. Shelters are required to determine a cat’s socialization level, or comfort level, with humans in order to decide what disposition options are available for that cat. Making this determination quickly is beneficial to the cat’s well-being by shortening shelter stay and improving opportunities for live release [7,8,9]. Making this determination is difficult for a variety of reasons; whether the cat was owned or unowned, primarily indoor or outdoor, free-roaming with a caregiver or completely self-reliant are all factors that likely could influence a cat’s behavior. In addition, the shelter environment is typically highly stressful and can result in cats behaving in uncharacteristic ways. Highly socialized and adoptable cats sometimes display fearful or aggressive behavior when under stress which may not be obviously different than a feral cat [10]. High stress levels can also result in inhibition of normal behaviors when confined to a cage [11]. 

Cats’ socialization with humans forms a spectrum from truly feral to well-socialized, and cats can change where they are along that spectrum with time and/or behavioral rehabilitation [10,12]. Truly feral cats that were not socialized with people while kittens will remain wary of humans throughout their lives [10]. Despite the importance of differentiating between moderately- to well-socialized but frightened cats from cats that are truly feral, there are currently no validated methods of assessing or categorizing cats upon intake to an animal shelter. Nor is there much published research on defining socialization status by behavioral cues; published research on cat socialization has been concerned with behavioral development and how early handling of kittens can influence their responses to humans [11,13]. Therefore, a measurement instrument is needed that could be used with both socialized and unsocialized cats by their owners or caregivers to define and describe a cat’s individual level of socialization in his/her normal environment.

This study is the first of a three-phase project to develop a reliable and valid assessment tool that shelter staff can use to determine which cats are truly feral and which are frightened but socialized to humans in a shelter setting. The second phase developed and evaluated the practicality and potential for a variety of in-shelter assessments to indicate the socialization level of a cat within three days of intake [14]. The third phase used the survey described here in conjunction with the results from the in-shelter assessments to measure the efficacy of our structured assessments in predicting the actual socialization levels of cats [15]. 

The first phase charts the development of a Cat Behavior and Background Survey. We designed a survey in which owners of pet cats or caregivers of foster or feral cats (to be hereafter referred to collectively as “caregivers”) reported the cats’ behavior towards people in the cat’s normal environment. Important requirements in developing this survey were that it is applicable to indoor and outdoor cats, easy to understand and useable by non-experts, as well as show acceptable levels of reliability and validity.

In order for any survey to be useful, it must provide reliable and valid data. The concept of reliability is simple: the survey instrument must measure its outcome in a reproducible fashion [16]. Two primary characteristics of a reliable instrument are stability, which is based on the reproducibility of the survey on different occasions by different people (test-retest agreement), and internal consistency, which is based on a single administration. The assessment of validity provides information about how well the survey instrument measures what it was intended to measure. No other published analyses of similar survey instruments were found by the authors. Therefore, we have used a variety of methods to estimate the validity of this survey. This article describes the development of the survey and examines its performance based on inter-rater reliability, test-retest agreement and internal consistency, as well as estimates of construct and criterion validity. This study was approved by the ASPCA^®^ Institutional Review Board. 

## 2. Methods

### 2.1. Overall Survey Design and Revision

The Cat Behavior and Background Survey was designed so that any person familiar with the cat could rate the cat’s socialization to humans in the cat’s usual environment based on their knowledge of the cats typical behavior. The survey needed to be effective in measuring the behavior of indoor or outdoor cats, socialized or unsocialized cats, as well as owned pet cats or free-roaming cats with one or more caregivers. The resulting ratings had to reflect the range of socialization found in cats (or fear/comfort) with humans, from “extremely frightened of humans and unaccustomed to their close proximity” to “extremely accustomed to interacting with humans and very comfortable with them”. To accomplish this, a series of 13 questions asked caregivers to rate frequency of cats’ behaviors with them on a scale of 0 to 10 (with anchors from “Never” to “Always”, plus an option of “I don’t know/have never tried”). In designing the questions, the focus was on variables that were as objective as possible, and also could be answered by someone with limited cat behavior knowledge. Also considered was how the behavior of cats might be different during routine activities that include interactions with familiar people and places as compared to novel people or situations. 

The survey was created by a subset of the authors: a shelter veterinarian, two certified applied animal behaviorists, an experienced shelter professional, and a veterinary epidemiologist. Questions were derived from the collective professional experience of the authors as well as a post-adoption questionnaire used during the development of the Meet-your-Match^®^ Feline-ality™ program [17], published behavior assessments [18,19] and research on the reactions to stress by cats [7,20,21,22]. The Cat Behavior and Background Survey (Appendix) requested the caregiver’s contact information, the amount of time the person had known the cat, and information about the cat including ratings of behavioral tendencies in a variety of situations. The survey was specifically designed to assess the socialization level of a cat and was not designed to determine the adoptability or the level of aggression of a cat. Questions were pilot-tested in February 2010 on 29 cats with seven cat caregivers and revised for clarity as necessary. The survey was then used in a study of 250 cats from April to October 2010 at the Humane Alliance Spay/Neuter Clinic in Asheville, North Carolina, United States (HA). Based on these results, we revised the Cat Behavior and Background Survey again to attempt to gain better insight into the cats’ behavior by separating the response option “I don’t know/have never tried” into two options: “I don’t know” and “It is not safe to try”. A summary of the different components of the reliability and validity including the locations used and numbers of cats is shown in Table 1.

**Table 1:** A summary of the components of this reliability and validity study including where, when and how many cats were included.

Study Component	Date	Facility	Type of Facility	Number of cats
Inter-rater and test-rest agreement	June–August 2011	Tabby’s Place; Feline Freedom Coalition	Sanctuaries	54 cats at Tabby’s Place 31 cats at Feline Freedom Coalition
Internal consistency	April–October 2010	Humane Alliance	Spay/neuter clinic	250 cats
Survey Pilot used for Construct validity	February 2010	Humane Alliance	Spay/neuter clinic	29 cats
Criterion validity	June 2011	Tabby’s Place	Sanctuary	15 cats (a subset of the 54)

### 2.2. Location Selection

For the inter-rater and test-retest reliability, we needed to have multiple caregivers who knew the cats well and could complete the Cat Behavior and Background Survey on two different occasions. Therefore, we identified two cat sanctuaries where cats stayed as residents for months or years and who cared for cats ranging widely in level of socialization to people, from very unsocialized to highly socialized. Criterion validity was also evaluated at a sanctuary since we needed cats to be available for video recording. Both sanctuaries selected were known to maintain high standards of animal care. One sanctuary was Tabby’s Place in Ringoes, New Jersey, United States, a sanctuary/adoption center that housed about 95 cats. Multiple staff and volunteer caregivers knew the cats very well. Cats were housed in indoor group rooms, most of which had access to an outdoor enclosed patio. Most indoor rooms had a large window onto the lobby. The second sanctuary was Feline Freedom Coalition in Ravenel, South Carolina, United States, housing about 150 cats, the majority of which were relatively unsocialized to humans. The cats lived in three compounds, each of which had an outdoor area (30 × 60 feet) and an indoor climate controlled area. 

To be eligible for study inclusion, sanctuary cats had to be 6 months to 10 years old (by best estimate), not seriously health or mobility impaired, not pregnant, nursing or in heat, housed at the sanctuary for at least one month and seemingly unlikely to be adopted during the month between the two survey administrations.

In order to have a large enough sample size for factor analysis, caregiver surveys from the 250 cats HA study were used for assessing internal consistency. In addition, the pilot study at HA was designed to examine construct validity using the extreme groups approach [16]. 

### 2.3. Calculation of the Overall Socialization Score

The 13 survey questions which asked caregivers to rate the frequency of cats’ behaviors on a scale of 0 to 10 were designed to also develop a single overall Socialization Score. This was done by calculating the median of these 13 behavior ratings questions. Determining which of these 13 questions to keep in the overall Socialization Score was decided after examining the reliability and validity of the individual questions as well as the overall score with and without the specific questions of concern. 

### 2.4. Participants for Inter-Rater Reliability and Test-Retest

No human demographic data were collected; based on names and researcher presence where the study was conducted, we knew there was a mix of male and female caregivers with owners or caregivers required to be greater than 18 years of age. 

For inter-rater reliability and test-retest, the survey was completed by staff and volunteers in the two sanctuaries (May through August 2011). Management at each location agreed to have two caregivers complete the Cat Behavior and Background Survey for each cat and for each caregiver to repeat the survey for the same cats, one month later. All caregivers were asked to complete the survey independently and confidentially within a 7 to 10 day window for each survey repetition. All caregivers were expected to use their knowledge of their usual interactions with these cats to complete the survey and not cause undue stress to the cats or put themselves at risk.

At Tabby’s Place, 36 different male and female staff and volunteer caregivers completed the survey for 54 cats, with 52 unique cat-caregiver pairings that were used for analysis of inter-rater reliability so that each cat was included in this analysis only once. For test-retest agreement, the data were included once for each cat where the same person had completed two surveys about 1 month apart. Therefore, there were 36 unique caregivers and one duplicate cat. These 36 pairs were then used for the test-retest calculations. At Tabby’s Place, the first assessment was done between May 11, 2011 and May 21, 2011; the second assessment was between June 15, 2011 and July 10 2011. The time between the first and second assessment ranged from 28–56 days, with a median of 35 days.

Feline Freedom Coalition had two female staff caregivers who were familiar with all the cats at that sanctuary. These two caregivers rated the same set of 31 cats in both time periods. Here, the first observer rated cats between July 6, 2011 and July 29, 2011 and again between August 10, 2011 to August 22, 2011. The time between the first and second period for the first observer ranged from 12–47 days with a median of 28 days. The second observer rated cats between July 1, 2011 and July 24, 2011 and again between August 22, 2011 and August 24 2011. The time between first and second period for the second observer ranged from 31–52 days with a median of 52 days.

### 2.5. Data Analyses for Inter-Rater Reliability and Test-Retest

Spearman rank sum correlation coefficients and 95% confidence intervals were used to analyze all ordinal categorical responses for the 13 questions as well as the overall Socialization Score. Standard statistical software was used for these analyses (StataSE 12, StataCorp LP, College Station, TX, USA). For all questions, responses were kept as the number circled by the respondents and ranged from 0 (least socialized) to 10 (most socialized). Appendix 1 includes all of the questions. 

For inter-rater agreement, “I don’t know” was considered missing since different people would be expected to have somewhat different knowledge of the cats. “It’s not safe to try” was considered to be a valid answer for a cat based on the cat’s behavior. For test-retest agreement, the responses “I don’t know” or “It’s not safe to try” were included as separate valid answers choices since we had hoped that the caregivers would be able to answer questions with the same responses for both survey repetitions. To provide a numerical response for “It’s not safe to try”, −1 was used and for “I don’t know”, the numeral 11 was used.

### 2.6. Participants for Internal Consistency Analysis

For the internal consistency analysis, a large data set was needed, so the survey data collected from the HA study were used. The owners or caregivers at HA were recruited through professional networking, an ASPCA^®^ member regional news alert, flyers, phone conversations with callers to HA’s scheduling desk, and newspaper advertisements and articles about the study (February 2010 and April 2010 through October 2010). 

### 2.7. Data Analysis for Internal Consistency: Exploratory Factor Analysis

The 13 survey questions that were scored from 0 to 10 from the HA caregivers were examined using factor analysis to determine if they all appeared to be measuring the same underlying concept (sociability). Correlations among responses to all 13 questions were calculated and very high (>0.8) and very low (<0.3) correlations were evaluated to determine if they should be excluded from the factor analysis [23]. The number of factors to extract was decided using both an eigenvalue >1.0 and examination of the scree plot. Bartlett’s test of sphericity (to support that there was a relationship among the items) and Kaiser-Meyer-Olkin Test (to support that the variables share common factors; >0.7 was needed) were examined [23]. As needed, the factors were rotated trying three orthogonal and three oblique rotations to create a clear pattern of loadings. If a single question did not load with others, it was removed and the factor analysis continued. Cronbach’s alpha [23,24] was calculated after the final factors were selected and the influence of removing each variable was examined in conjunction with the number of missing data points to see if any variables could be omitted. Patterns of the factors were evaluated to examine how the survey questions might be related. The variables that did not load well with others and that had low reliability were considered for exclusion in calculating the overall Socialization Score. These analyses were done in IBM SPSS Statistics 20 (1 New Orchard Road, Armonk, NY, USA).

### 2.8. Analysis of Survey Validity

There were no established, published standards for evaluating socialization level of cats, against which our survey results could be compared. In addition, we recognized that a cat’s behaviors may change over time, which can make evaluating the cat’s true socialization status even more difficult. Within these constraints we examined two aspects of the validity of the survey: construct validity and criterion validity.

### 2.9. Construct Validity Analysis

We used extreme groups validation for this component [16]. During the pilot testing on 29 cats and seven caregivers, owners and caregivers were asked to bring in the most socialized and the most unsocialized cats to which they had access and complete the Cat Behavior and Background Survey for that cat. We were therefore expecting to see caregiver Socialization Scores from the survey tending to be at either end of the socialization spectrum.

### 2.10. Criterion Validity

It is possible that cat caregivers’ interpretation of cats’ behavior was different from that of the “gold standard” of expert opinions, which would affect interpretation of the results of the survey. In order to determine agreement between caregivers and experts, criterion validity was assessed using nine video clips for each of 15 cats interacting with their caregivers. These cats had Socialization Scores, using the Cat Behavior and Background Survey, ranging from 0 to 10 with a median of 7. The caregivers were asked to attempt to interact with the cats in specific ways which reflected the interaction described by the Cat Behavior and Background Survey questions while video recording. Video was recorded by 14 different caregivers at Tabby’s Place who wore a GoPro mini video camera on their chests while interacting with a cat, while they filled out a modified series of questions about the cat’s behavior. The caregivers were asked to attempt nine versions of the original survey questions: Approach Within Two Feet, Pet the Cat, Pick Up the Cat, Plays with the Cat using a Toy, invite the cat to Approach for Affection, see if the cat would Stay Near them while they walked around, and to note if the cat Meowed, hissed, spit, growled, swatted or bit at any time, and if the Cat was Slinking/Crouched or was Tense at any time. The caregivers were instructed not to perform interactions with the cats that would place them at risk or excessively stress the cats. The caregivers who interacted with the cats and the experts who viewed the videos of these interactions independently completed a questionnaire indicating whether or not each of these behaviors was tolerated by/displayed by the cat (“yes” or “no”). Responses of “yes” indicated more socialization; the more “yes” responses the more socialized the cat. The independent experts also provided a subjective global Socialization Score of 0 (extremely unsocialized) to 10 (extremely socialized).

The caregivers did not have knowledge of the cats Socialization Score. The independent cat experts did not have knowledge of the cats’ Socialization Score or caregiver responses on the videos. The percent of “yes” responses out of the videos evaluated was calculated and compared between the caregiver and the median percent of “yes” of the experts as well as to the subjective global socialization score given by the experts. Stata statistical software was used for this analysis. 

## 3. Results

### 3.1. Inter-Rater Reliability and Test-Retest

Tabby’s Place included 36 caregivers and 52 different pairs of raters compared to the two caregivers and 31 cats analyzed at Feline Freedom Coalition. Tabby’s Place showed modest or fair agreement on many questions (see Table 2). Inter-rater reliability was less than 0.50 for Come Within Two Feet while Eating, Very Active and Stays Near. Comes Within Two Feet Other Times, Meows, Plays with Toys, Tends to Slink/Crouch or be Tense and Settles Quickly when Startled had inter-rater correlations between 0.51 and 0.60. Test-retest showed agreement below 0.50 when “don’t know” and “it’s not safe to try” were included as data points for Come Within Two Feet while Eating and Settles Quickly when Startled. For both variables, omitting the “don’t know” and “it’s not safe to try” responses improved agreement to >0.60. Omitting the “don’t know” and “it’s not safe to try” responses from any variable with >2 of these responses improved agreement; for some variables the improvement was substantial (Table 2). 

Feline Freedom Coalition (two individuals did the same set of cats twice) showed very high levels of inter-rater and test-retest agreement for all questions and the overall socialization score. The median inter-rater agreement for the individual questions was 0.927 (range of 0.790–0.957). The median test-retest correlation for each question for person 1 was 0.988 (range of 0.880–0.999) and for person 2, 0.920 (range 0.819–0.943). The overall Socialization Scores with all 13 questions for test-retest and inter-rater correlations were between 0.873 and 0.988 and for the 11 question Socialization Score (without Plays with Toy or Very Active) were between 0.855 and 0.980. For Meows at Person, Very Active, Plays with Toys, Settles Quickly and overall Socialization Score, the inter-rater score was slightly lower than the test-retest scores. 

**Table 2:** Agreement for the Caregiver Survey Data for Inter-rater Reliability and Test-Retest for Tabby’s Place with 52 unique pairs of caregivers and 52 different cats (inter-rater reliability) and 36 caregivers on 35 different cats (test-retest).

Question	Person 1 Repetition 1	Person 2 Repetition 1	Inter-rater Agreement	Person Repetition 1	Person Repetition 2	Test re-test Agreement	Test-retest Agreement if Missing or Don’t Know answers were > 2 responses for the question
**Come within 2 feet while eating **			0.211 on 35 observations (95% CI: −0.132 to 0.508)			0.360 on 36 observations (95% CI: 0.035 to 0.616)	0.670 on 25 observations (95% CI: 0.374 to 0.842)
Median score	10	9		10	9		
It’s not safe to try	0	0		0	1		
Missing/don’t know	9	9		7	7		
**Come within two feet other times**			0.524 on 52 observations (95% CI: 0.293 to 0.697)			0.639 on 36 observations (95% CI: 0.393 to 0.799)	
Median score	10	10		9	10		
Missing/don’t know	0	0		0	1		
**Allows petting**			0.813 on 49 observations (95% CI: 0.690 to 0.891)			0.886 on 35 observations (95% CI: 0.784 to 0.941)	
Median score	8	9		9	9		
It’s not safe to try	0	2		1	1		
Missing/don’t know	2	0		1	2		
**Allows holding**			0.680 on 26 observations (95% CI: 0.398 to 0.845)			0.795 on 36 observations (95% CI: 0.632 to 0.891	0.964 on 24 observations (95% CI: 0.917 to 0.984)
Median score	1	0		4	5		
It's not safe to try	3	5		3	3		
Missing/don’t know	12	16		8	7		
**Meows at person**			0.519 on 50 observations (95% CI: 0.281 to 0.697)			0.671 on 36 observations (95% CI: 0.439 to 0.819)	
Median score	0	1		1	3		
Missing/don’t know	2	0		1	0		
**Is very active**			0.460 on 50 observations (95% CI: 0.209 to 0.655)			0.937 on 36 observations (95% CI: 0.879 to 0.968)	.
Median score	4	4		4	5		
Missing/don’t know	2	0		0	1		
**Approaches for affection**			0.709 on 51 observations (95% CI: 0.538 to 0.823)			0.789 on 36 observations (95% CI: 0.622 to 0.888)	
Median score	5	5		6	6		
Missing/don’t know	1	0		0	0		
**Stays near**			0.425 on 52 observations (95% CI: 0.172 to 0.626)			0.651 on 36 observations (95% CI: 0.410 to 0.807)	
Median score	7	7		7	8		
Missing/don’t know	0	0		0	0		
**Plays with toys**			0.511 on 32 observations (95% CI: 0.198 to 0.730)			0.570 on 35 observations (95% CI: 0.292 to 0.759)	0.912 on 25 observations (95% CI: 0.808 to 0.961)
Median score	5	5		5	6.5		
Missing/don’t know	12	14		8	8		
**Tends to slink (body low to ground while moving), crouch while moving or be tense **			0.562 on 49 observations (95% CI: 0.334 to 0.728)			0.736 on 35 observations (95% CI: 0.533 to 0.858)	
Median score	0	0		1	0		
Missing/don’t know	1	2		2	0		
**Settles quickly when startled**			0.520 on 28 observations (95% CI: 0.182 to 0.748)			0.425 on 35 observations (95% CI: 0.107 to 0.664)	0.615 on 21 observations (95% CI: 0.250 to 0.827)
Median score	9	8		8	7		
Missing/don’t know	9	18		12	8		
**Lets unfamiliar people approach**			0.750 on 33 observations (95% CI: 0.547 to 0.869)			0.517 on 35 observations (95% CI: 0.222 to 0.725)	0.804 on 26 observations (95% CI: 0.605 to 0.909)
Median score	7	8		8	8		
Missing/don’t know	9	11		6	8		
**Lets unfamiliar people pet**			0.839 on 26 observations (95% CI: 0.669 to 0.926)			0.593 on 34 observations (95% CI: 0.318 to 0.775)	0.950 on 24 observations (95% CI: 0.886 to 0.978)
Median score	6	6		7	7		
It's not safe to try	1	3		1	0		
Missing/don’t know	14	12		8	9		
**Overall Sociability Score all 13 variables **			0.763 on 52 observations (95% CI: 0.619 to 0.857)			0.831 on 36 observations (95% CI: 0.691 to 0.911)	
Median score	7.5	7		8	8		
**Sociability Score without toy or active**			0.705 on 52 observations (95% CI: 0.536 to 0.820)			0.818 on 36 observations (95% CI: 0.669 to 0.904)	
Median score	8	8		8	8		

### 3.2. Internal Consistency

The responses “don’t know” and “it’s not safe to try” were considered to be missing and were excluded. Plays with Toy was excluded from this analysis because of the lower reliability at Tabby’s Place and because it was originally included primarily to find out if these cats had experience with interactive toys. We hypothesized at the time the survey was designed that knowing the cats experience with toys could be useful in interpreting the assessment which involved playing with toys. The remaining 12 variables were included which resulted in 226 observations for the factor analysis. The correlations among the 13 variables ranged from 0.25 to 0.91 with 5 greater than 0.8 (Come with 2 feet while Eating and Come within 2 Feet Other Times; Come within 2 Feet Other Times with Allows Petting; Allows Petting with Allows Holding; Allows Petting with Approaches for Affection; Lets Unfamiliar People Approach with Unfamiliar People Pet) and 1 less than 0.3 (Very Active with Tends to Slink/Crouch or be Tense). These six correlations did not appear to be a problem in the factor analysis. 

Two factors were extracted using principal component analysis, based on the scree plot and cumulative percent variance (76%). However, this was due to the variable Very Active loading by itself. Because we were interested in how the other variables were related, we began again without the Very Active variable. Without Very Active, all remaining variables loaded on a single factor with an initial eigenvalue of 8.005 and percent variance of 72.8%. Communalities for the 11 final variables ranged from 0.48 (Meows at Person) to 0.864 (Allows Petting). The loadings on the single component are shown in Table 3. Bartlett’s test of sphericity was 0.000 indicating that the correlation matrix was not an identity matrix and the Kaiser-Meyer-Olkin Test was 0.924 indicating that sampling adequacy was “marvelous” [23]. Cronbach’s alpha was 0.962. The change in Cronbach’s alpha with removing a single item ranged from 0.955 (for Allows Petting and Approaches for Affection) to 0.963 (for Meows at Person). Due to these results and because some of these questions could have high numbers of missing responses, all of these 11 variables were kept and used in the final Socialization Score.

**Table 3:** Factor Loadings from the Component Matrix for 11 Questions in the Survey with Principal Component Analysis.

	Component
	1
Come within 2 ft – eating	0.833
Come within 2 ft – other times	0.921
Allows petting	0.930
Allows holding	0.893
Meows at person	0.693
Approaches for affection	0.916
Stays near	0.910
Reverse coding slink/crouch/be tense	0.723
Settles quickly when startled	0.823
Lets unfamiliar people approach	0.836
Lets unfamiliar people pet	0.870

### 3.3. Construct Validity (Extremes of Scores for Pilot)

For this analysis, the caregivers’ Cat Behavior and Background Survey results showed 45% of cats with Socialization Scores of 0 or 1, 10% with Socialization Scores of 4 or 5 and 45% with Socialization scores of 7 and above (20% at score 10). When the final version of the Socialization Score (Without Toy and Very Active) was used, 48% were scored 3 or less, 7% were scored 4 and 45% were 7 and above. 

### 3.4. Criterion Validity

There were five cats with more than one question where the caregiver score for that cat and the experts’ score did not agree from the video (one replied with a yes and one a no which resulted in a greater than 11% difference). The cat with the greatest difference in percent yes between the caregiver and expert was described by the experts as “unhappy with that caregiver”; the percent yes from the experts was 25% and the percent yes from the caregiver was 44%. This cat also had a median global expert score of 5 and a Socialization Score of 0. Another of these five cats appeared to be in pain according to the experts and uncomfortable around the other cats currently in the housing area; several experts commented on this. She was scored as 67% “yes” by the caregiver and 80% “yes” by the experts with a median global score of 8 and Socialization Score of 4.5. Another cat had some video difficulties with poor sound and in one instance a poor view of the cat; this cat was scored 11% “yes” by the caregiver and 25% “yes” by the expert (median global score of 2). One cat was observed to swat, hiss and growl at the caregiver and scored with more “yes” responses by the experts than the caregiver (46% “yes” *vs.* 33% “yes”, respectively). This cat also had a large difference between the median global score of the experts and the Socialization Score (5 *vs.* 0, respectively). The final cat was scored as 89% “yes” by the caregiver and 76% “yes” by the experts; the experts commented he seemed a bit anxious in the beginning of the interaction.

## 4. Discussion

Because test-retest data compares the same person’s responses it is expected to be higher than inter-rater reliability [16]. If there was a difference between inter-rater and test-retest correlations, inter-rater correlations were always lower. Feline Freedom Coalition had only two people which would tend to decrease the variability and increase the magnitude of the correlations; this situation would be expected to show maximum levels of agreement. Tabby’s Place illustrates the more realistic situation with 36 different people rating 35 different cats and did show more modest correlations than Feline Freedom Coalition. The level of agreement considered to be adequate using correlations is often subjective. We believed that correlations over 0.60 were good and those over 0.80 were excellent. We hypothesize that inter-rater correlations <0.60 could be due to some individual observers having more limited knowledge of cat behavior than others or that some cats had different relationships with different observers and there were true differences. Our efforts to gain more information when the caregivers could not rate a cat on a question by dividing “don’t know” into two components (“it’s not safe to try” and “I don’t know”) failed. Our belief that caregivers should be able to consistently indicate the difference between circumstances when they have not seen the cat in that situation or they never tried to interact with the cat in that situation due to safety concerns was not supported by the data. In fact, removing both responses substantially improved test-retest agreement for questions where two or more responses fell into those categories. It is possible that the wording we chose for these two response options was poor. Without further work to better elucidate the effect of language choice, we recommend that a simple “I don’t know” be used whenever a person cannot adequately score a cat on one of the 11 questions. 

Constructs are the proposed underlying factors that result in characteristics we can observe [16]. Therefore, our survey attempted to tap into our underlying construct of socialization and create a way to measure this. Internal consistency was quite high with the final 11 questions (excluding Plays with Toy and Very Active), indicating that our questions that used a rating of 0 to 10 were all evaluating a single underlying construct. Removing any single question did not improve the Cronbach’s alpha in a meaningful way. 

Due to a lack of an objective measure of cat socialization, we used the caregivers’ own determinations of very unsocialized and very socialized cats to develop an estimate of construct validity. The extreme groups assessment, while using a small sample, does support the validity of our survey because most of the cats had Socialization Scores at each end of the range. In addition, the two cats who were not at the extreme ends (less than or equal to 3 or greater than or equal to7) were right on the cusp at a Score of 4. We recognize that construct validation is an ongoing process; papers reporting on the second and third part of this project [14,15] provide some additional application of this survey to a new set of owners and caregivers and their cats.

Criterion validity is usually defined as the correlation of the new survey with some other measure, ideally one that is considered to be a “gold standard” for the trait by experts in the discipline. However, in our case there was no gold standard. We elected to use a synthesis of expert opinions to provide an approximation of a gold standard, recognizing that this is still not fully objective. To make this as objective as possible, we designed the questions to be answered by the videos to be very clear and unambiguous and the experts were blinded to the cats’ Socialization Scores and caregiver responses. There was some disagreement between the experts and the caregiver. The cats where disagreement was seen had both low and high Socialization Scores which appeared to indicate that this disagreement was not strongly related to level of socialization. We found that compared to the experts, the caregivers tended to score cats with aggression or pain as less socialized to humans. This could be an inherent limitation in the survey or be related to the direct impact of the caregiver handling an aggressive cat. In addition, the difference between the live interaction (where all aspects of the cat are easier to see) and viewing the recorded interaction could also have influenced the results. Experts commented that one cat seemed unhappy with that caregiver and that another cat was anxious at first about the interactions. These perceptions by the experts could lead to differences with the caregiver, where a caregiver could have a bias due to previous interactions with that cat. Since Tabby’s Place caregivers volunteered to participate in this project the only requirement was that they knew the cat well. We did not specify what their relationship with the cat should be. Also, while we attempted to select clear videos where the cat was visible at all times, this was not always possible for every moment of every video clip. This made some experts question their responses, particularly for one video clip.

It is important to note that the caregivers, especially those interacting with cats who avoided human interaction, had to develop and record their survey responses based on the interactions they were able to have with the cats. The amount of space the cat has likely influences the interactions as well as the responses of the caregivers. Therefore, there is probably a limitation to the caregiver survey in that some small subset of cats may avoid human contact when possible, but when in a more confined space may display social behavior. 

We believe that the overall Sociability Score shows acceptable inter-rater and test-retest reliability when Plays with Toy or Very Active are not included. We realize that how strong the agreement should be is a somewhat subjective decision. Our continued work with the Socialization Score in the other two papers for this project [14,15] supports its usefulness. 

## 5. Conclusions

The survey described here can be used by a variety of people in contact with individual cats, including those with little or no background in cat behavior. We believe that it is sufficiently reliable and valid to be used for our purposes. The survey can be used to describe the socialization level of cats to humans when the cats are in a variety of situations in their normal environment. We also think that this survey might be useful to others in exploring cats’ behaviors in their typical environment. A key element for the survey to be as reliable as possible is for the person completing the survey to know the cat well (at least a month or more) and preferably to have observed the cat in a variety of usual as well as novel situations. When a person using the survey cannot accurately score the cat on a question, a simple “I don’t know” should be used and that response treated as missing when the Socialization Score is calculated.
